# Substance Use Disorders in Men Presenting to a Psychosexual Clinic

**DOI:** 10.1155/2014/486383

**Published:** 2014-01-06

**Authors:** Ravi Philip Rajkumar

**Affiliations:** Department of Psychiatry, Jawaharlal Institute of Postgraduate Medical Education and Research (JIPMER), Dhanvantari Nagar, Pondicherry 605 006, India

## Abstract

*Introduction*. Substance use disorders (SUDs) are commonly associated with a variety of psychiatric disorders. Community-based studies have found a significant association between SUDs and sexual dysfunction in men, with a possible causal relation in the case of nicotine. *Methods*. The case records of 105 men presenting to a clinic for patients with psychosexual disorders were reviewed. Men with and without comorbid SUDs were compared in terms of demographic, clinical, and familial variables. *Results*. 25 of the 105 men (23.8%) had a lifetime diagnosis of SUD, and 19 (18.1%) had a current SUD. The commonest substances involved were nicotine (*n* = 21, 20%) and alcohol (*n* = 9, 9.5%). Men with comorbid SUDs were more likely to report a family history of substance dependence, particularly alcoholism. Single men with SUDs were more likely to have a comorbid mood disorder. *Conclusion*. SUDs, particularly nicotine and alcohol use disorders, are common comorbidities in patients with psychosexual disorders. Identifying and treating these disorders in this population are important aspects of management.

## 1. Introduction

Substance use disorders (SUDs) are a global health problem which present in a variety of clinical settings. Substance use may complicate an underlying medical or psychiatric disorder and can also lead to medical or psychological morbidity in its own right. High rates of substance use have been reported in patients with psychiatric syndromes such as schizophrenia [1], mood disorders [2, 3], and anxiety disorders [3]. Patients with both conditions, sometimes referred to as “dual-diagnosis” patients, have been found to have more severe symptoms and poorer outcomes [1, 4].

Psychosexual disorders, also termed psychogenic sexual disorders, are dysfunctions of the sexual response cycle that cause impaired sexual performance and distress to affected patients and their partners. These disorders can affect any phase of the sexual response cycle. The commonest sexual disorders seen in male patients are premature ejaculation [5] and erectile dysfunction [6]. Earlier studies have reported significant rates of problematic substance use in men with sexual disorders [7–9], but this association has been less well studied than in patients with other mental disorders, and its causal significance is unclear. A consistent association between erectile dysfunction and tobacco use has been found [7, 9], but the effects of alcohol use on sexual dysfunction are less clear [10]. However, this association has not been systematically examined in the Indian context. In the current study, we retrospectively examined the rates of substance use, abuse, and dependence in young and middle-aged South Indian men presenting to an outpatient clinic for psychosexual disorders.

## 2. Materials and Methods

The case records of 105 male patients who attended the Marital and Psychosexual Disorders (MAPS) Clinic at the Jawaharlal Institute of Postgraduate Medical Education and Research (JIPMER) between January 2011 and October 2013 were reviewed. JIPMER is a tertiary care institute located in Pondicherry, South India. Alcohol use disorders are a significant problem in Pondicherry, which was a further motivation for conducting this study.

The majority of clinic attenders are young men, both married and single. Patients are referred to the clinic either by self-referral or from other departments in JIPMER, particularly general surgery and urology. At initial presentation, a brief assessment is made by a trained psychiatrist, and an appointment is given for a detailed evaluation in the MAPS clinic. On the day of this appointment, patients are evaluated using a semistructured interview to elicit a sexual history as well as details of medical and psychiatric comorbidity, family history of mental illness, and early childhood experiences. All interviews are conducted by psychiatry residents, and diagnoses are made according to ICD-10 clinical guidelines after review by a consultant. Following diagnosis, patients received psychological interventions, including education and behaviour therapy. Pharmacotherapy in the form of antidepressants and anxiolytics was used for the management of comorbid anxiety and depression, and the short-acting serotonin uptake inhibitor dapoxetine was used in selected patients with premature ejaculation.

Statistical analyses were carried out using the WinPEpi version of the OpenEpi program. All tests were two-tailed and a significance level of 0.05 was considered statistically significant.

## 3. Results

The sample consisted of 105 men. Of these, 25 (23.8%) had a lifetime diagnosis of a substance use disorder; a further 7 (6.7%) had used alcohol or nicotine but were not dependent on them. Of the 25 with an identified substance use disorder, 3 only had a harmful use pattern without dependence; all of these three men were using alcohol. The remaining 22 (21%) fulfilled criteria for substance dependence: 21 had nicotine dependence, and 6 had alcohol dependence. Five men had both nicotine and alcohol dependence. One patient had harmful use of benzodiazepines, and one reported harmful use of cannabis. Among those men with a history of alcoholism (*n* = 6), the mean age at onset of dependence was 22.8 ± 3.11 years; three of these men (50%) had an early (<25 years) onset. Only one of the men with alcohol dependence had a family history of alcoholism.

Of these 25 men, 19 (18.1%) fulfilled ICD-10 criteria for current harmful use or dependence: 17 with nicotine dependence and two with alcohol dependence, one with harmful use of alcohol and one with harmful use of benzodiazepines. The two men with current alcohol dependence also had active nicotine dependence. The remaining six had discontinued substance use prior to receiving a diagnosis of sexual dysfunction.

When patients with comorbid substance use were compared with those without, the two groups were comparable on most variables, including age, duration of illness, types of sexual dysfunction, comorbid conditions, and early childhood adversity. However, men with comorbid substance use were more likely to have a family history of substance dependence (alcohol or nicotine) in a first-degree relative (14/25 versus 23/80; *χ* ^2^ = 6.198, *P* = 0.013). This association held good even when only family histories of alcoholism were considered (11/25 versus 18/80; *χ* ^2^ = 4.404, *P* = 0.036). Anxious-avoidant personality was more common in men without substance use disorders (15% versus 4%), but this difference was not statistically significant (Table 1). 

When the subgroup with active substance use alone (*n* = 19) was compared with the group without substance use disorders, they were also significantly more likely to have a family history of substance dependence (12/19 versus 23/80; *χ* ^2^ = 7.953, *P* = 0.005) or alcoholism in particular (9/19 versus 18/80; *χ* ^2^ = 4.787, *P* = 0.029). However, they did not differ on any other clinical or demographic variable, including the type of sexual dysfunction experienced.

Separate analyses were then conducted to compare the profiles of single and married men with and without substance use disorders. Among single men, patients with comorbid substance use disorders were more likely to suffer from a comorbid mood disorder (5/15 versus 4/51; *χ* ^2^ = 6.395, *P* = 0.011) and were more likely to have a family history of alcoholism (9/15 versus 11/51; *χ* ^2^ = 8.106, *P* = 0.004) than those without substance use disorders; the two groups were comparable in other respects.

Among married men, patients with comorbid substance use disorders were less likely to report infertility as a precipitating or aggravating factor for their sexual dysfunction (none out of 10 versus 9/29; *χ* ^2^ = 4.034, *P* = 0.045); however, they did not differ from married men without substance use disorder on any other clinical or demographic variable. Nonsubstance users were numerically more likely to report marital disharmony (55.16% against 30% of married substance users), but this difference did not reach statistical significance.

## 4. Discussion

The rate of comorbid substance use disorders in men with psychosexual disorders was 23.8%; in 21%, this amounted to dependence. These rates are slightly higher than those reported for mood or anxiety disorders in general [3] but lower than those reported for schizophrenia [1] or bipolar disorder [2], and they are comparable to the rates of regular smoking (27.2%) reported in Australian men with erectile dysfunction [9]. This suggests that substance use disorders may be an important comorbidity in men with sexual dysfunction and should be screened for and addressed in its own right.

In this sample, nicotine and alcohol were the most common substances abused. As these substances are licit and freely available, they are commonly used by young men in India. In contrast, the use of other drugs such as benzodiazepines and cannabis was infrequent in our patients. This pattern is consistent with epidemiological research on the relative frequencies of various substance use disorders in India [11]. The rates reported in our patients are somewhat lower than those reported from deaddiction centres in India [12], though they are higher than those documented in the general population [13].

The most significant difference noted between men with and without substance use disorders in this study was the presence of a family history of substance dependence, particularly alcoholism, in the former group. This is in line with current evidence on the genetics of alcoholism and nicotine dependence [12]. Further research is needed to examine the possibility that substance use disorders and psychosexual disorders may arise from a shared genetic liability.

Single men with substance use and psychosexual disorders were more likely to suffer from mood disorders in our study. This may reflect the well-documented association between depression and substance use [13, 14]. Caution is required in managing depression in this population, as antidepressants can themselves worsen sexual dysfunction in some patients [15].

As this study was retrospective, a causal association between substance use disorders and sexual dysfunction could not be established. Moreover, the “control” group consisted of men with a primary diagnosis of sexual dysfunction, making meaningful comparisons difficult. Current evidence suggests that smoking cessation can improve sexual dysfunction in men [16]. Though a meta-analytic review found that the association between alcohol use and erectile dysfunction was inconsistent [10], an Indian study found a significant link between alcohol dependence and sexual disorders [17]. Managing the comorbid substance use disorder in these patients may therefore lead to resolution of the patient's sexual dysfunction.

The above results are subject to several limitations. Diagnoses were made using the ICD-10 clinical guidelines and depended on clinical skill rather than the use of a structured diagnostic interview. The retrospective design limits the generalizations that can be made from our findings. And finally, the study sample, which was drawn from a clinic for psychosexual disorders, may not accurately represent the young and middle-aged male population.

## 5. Conclusion

Substance use disorders are a common comorbid diagnosis in men with psychosexual disorders. In these patients, substance use disorders tend to run in families and may be associated with higher rates of mood disorder in single men. These findings have clear implications when assessing and treating men with sexual disorders. Further prospective hospital and community-based studies are needed to obtain a better estimate of the magnitude and correlates of this problem.

## Figures and Tables

**Table 1:** Clinical profile of men with psychosexual disorders, with (SUD+) and without (SUD−) comorbid substance use disorders.

Variable	SUD+ (*n* = 25)∗	SUD−^ ^ (*n* = 80)∗
Age	29.08 (6.1)	28.8 (7.49)
Duration of sexual disorder (in years)	2.71 (3.09)	3.2 (3.01)
Marital status		
Single	15 (60%)	51 (63.75%)
Married	10 (40%)	29 (36.25%)
Psychosexual diagnosis		
Erectile dysfunction	13 (52%)	30 (37.5%)
Premature ejaculation	6 (24%)	22 (27.5%)
Dhat syndrome	11 (44%)	37 (46.25%)
Impulse control disorder NOS	—	1 (1.25%)
Lack of sexual desire	—	1 (1.25%)
Ego-dystonic homosexuality	—	2 (2.5%)
Comorbid mood disorder	5 (20%)	10 (12.5%)
Depression	2 (8%)	6 (7.5%)
Dysthymia	2 (8%)	3 (3.75%)
Recurrent brief depression	—	1 (1.25%)
Mixed anxiety depression	1 (4%)	—
Comorbid anxiety disorder	4 (16%)	16 (20%)
Generalized anxiety disorder	3 (12%)	2 (2.5%)
Anxious-avoidant personality disorder	1 (4%)	12 (15%)
Specific phobia	—	1 (1.25%)
Anxiety disorder not otherwise specified	—	1 (1.25%)
Family history of mood disorder/suicide		
Mood disorder	—	6 (7.5%)
Suicide	1 (4%)	4 (5%)
Family history of substance dependence	14 (56%)	23 (28.75%)∗∗
Alcohol	11 (44%)	18 (22.5%)∗∗∗
Nicotine	3 (12%)	5 (6.25%)
Parental breakup or discord during childhood	6 (24%)	16 (20%)
Marital disharmony	3 (30%)^#^	16 (55.18%)^$^

^*^All figures are given as mean (standard deviation) or frequency (percentage).

∗∗Significant at *P* = 0.013.

∗∗∗Significant at *P* = 0.036.

^#, $^Calculated out of 10 married men in SUD+ and 29 married men in SUD−.
